# Development of a Genus-Specific Antigen Capture ELISA for Orthopoxviruses – Target Selection and Optimized Screening

**DOI:** 10.1371/journal.pone.0150110

**Published:** 2016-03-01

**Authors:** Daniel Stern, Diana Pauly, Martin Zydek, Lilija Miller, Janett Piesker, Michael Laue, Fred Lisdat, Martin B. Dorner, Brigitte G. Dorner, Andreas Nitsche

**Affiliations:** 1 Highly Pathogenic Viruses (ZBS 1), Centre for Biological Threats and Special Pathogens, Robert Koch Institute, Berlin, Germany; 2 Biological Toxins (ZBS 3), Centre for Biological Threats and Special Pathogens, Robert Koch Institute, Berlin, Germany; 3 Biosystems Technology, Institute of Applied Life Sciences, Technical University of Applied Sciences, Wildau, Germany; 4 Advanced Light and Electron Microscopy (ZBS 4), Centre for Biological Threats and Special Pathogens, Robert Koch Institute, Berlin, Germany; National Institute for Viral Disease Control and Prevention, CDC, China, CHINA

## Abstract

Orthopoxvirus species like cowpox, vaccinia and monkeypox virus cause zoonotic infections in humans worldwide. Infections often occur in rural areas lacking proper diagnostic infrastructure as exemplified by monkeypox, which is endemic in Western and Central Africa. While PCR detection requires demanding equipment and is restricted to genome detection, the evidence of virus particles can complement or replace PCR. Therefore, an easily distributable and manageable antigen capture enzyme-linked immunosorbent assay (ELISA) for the detection of orthopoxviruses was developed to facilitate particle detection. By comparing the virus particle binding properties of polyclonal antibodies developed against surface-exposed attachment or fusion proteins, the surface protein A27 was found to be a well-bound, highly immunogenic and exposed target for antibodies aiming at virus particle detection. Subsequently, eight monoclonal anti-A27 antibodies were generated and characterized by peptide epitope mapping and surface plasmon resonance measurements. All antibodies were found to bind with high affinity to two epitopes at the heparin binding site of A27, toward either the N- or C-terminal of the crucial KKEP-segment of A27. Two antibodies recognizing different epitopes were implemented in an antigen capture ELISA. Validation showed robust detection of virus particles from 11 different orthopoxvirus isolates pathogenic to humans, with the exception of MVA, which is apathogenic to humans. Most orthopoxviruses could be detected reliably for viral loads above 1 × 10^3^ PFU/mL. To our knowledge, this is the first solely monoclonal and therefore reproducible antibody-based antigen capture ELISA able to detect all human pathogenic orthopoxviruses including monkeypox virus, except variola virus which was not included. Therefore, the newly developed antibody-based assay represents important progress towards feasible particle detection of this important genus of viruses.

## Introduction

The genus *Orthopoxvirus* (OPV) (family *Poxviridae*, subfamily *Chordopoxvirinea*) comprises several species which are able to infect humans and animals alike. The most notorious member is variola virus (VARV), the causative agent of human smallpox. Fortunately, this devastating disease with mortality rates of up to 50% [1] was eradicated in 1977 [2]. Subsequently immunization with cross-protective vaccinia viruses (VACV) was ceased in particular due to rare but severe vaccination adverse events [3]. As a consequence, a growing proportion of today’s population is unprotected against infections by variola virus and other still circulating zoonotic OPVs, namely monkeypox virus (MPXV), cowpox virus (CPXV) and vaccinia virus (VACV) [4].

OPVs are immunologically cross-reactive large dsDNA viruses characterized by cytoplasmic replication [5]. OPV exist in two fully infectious virus forms enveloped by one (intracellular mature virion, IMV) or two membranes (enveloped virion, EV), each comprising a different set of membrane proteins [6]. However, only the abundant IMV form is extendedly stable in the environment, as the EV membrane is fragile and easily shed [7]. The IMV membrane contains more than 20 surface proteins with different, partly overlapping functions in the infection cycle of the virus, such as morphogenesis and transport, attachment to target cells, entry and fusion [5]. Attachment to target cells is mediated by interactions of three viral proteins with cell surface glycosaminoglycans (GAGs). The outer membrane proteins A27 and H3 of the IMV bind to heparin and heparan sulfate on target cells [8–11], while the protein D8 interacts with chondroitin sulfate [12]. For A27, the multimeric interaction with heparin is mediated at the heparin binding site (HBS) by the crucial KKPE-segment region located at the N-terminus of the trimer between amino acids 26 to 29 [13, 14]. Beside attachment, a large multi-protein complex composed of at least 12 transmembrane proteins mediates subsequent fusion of the viral and cell membranes [15, 16]. Among them, the membrane protein L1 is a prominent member as it induces potent neutralizing and protective antibodies [17–19].

Diagnosis of OPV infections cannot rely on clinical observations alone as several bacterial or viral agents cause clinical signs that closely resemble the early papular (e.g. Herpes simplex virus, *Bartonella henselae*) or late crustular (e.g. *Bacillus anthracis*) stages of an OPV infection [4]. Hence, diagnosis is usually based on the detection of viral particles by negative staining electron microscopy (EM) [20] or viral DNA analysis by (quantitative real-time) PCR (qPCR) [21]. While these methods are well-established and unsurpassed regarding assay speed (EM) or sensitivity and specificity (qPCR), they depend on expensive equipment and trained staff available only at specialized laboratories. However, the rising incidence of MPXV infections in rural Africa [22] as well as the potential deliberate release of VARV or MPXV in the case of a bioterrorist attack [23, 24] require settings where simpler antibody-based immunological diagnostics are available [25].

Until now, only one antigen capture ELISA for the detection of OPV has been described [26]. Highly sensitive detection of all OPV strains was achieved with a monoclonal capture antibody directed against a highly conserved antigenic structure on A27 and polyclonal rabbit anti-vaccinia/-mousepox virus detection antibodies. However, the development of an ELISA based solely on monoclonal antibodies (mAbs) for the detection of all OPV failed due to the lack of cross-reactivity of a second available mAb against mousepox virus (ECTV) and, more importantly, against the human pathogenic MPXV [27].

The goal of this work was to develop a mAb-based antigen capture ELISA able to detect all human pathogenic OPV. To this end, a two-stage target selection and screening strategy was employed. First, polyclonal antibodies (pAbs) against recombinant surface proteins A27, D8, H3 and L1 were generated and compared regarding their ability to detect VACV particles. Second, the protein inducing the most potent detection antibodies was used to generate mAbs, which were thoroughly characterized. The specificity and sensitivity for the detection of different OPV species isolates were tested for the best monoclonal-monoclonal antibody combination. Finally, the applicability of the newly developed antigen capture ELISA was successfully tested by the detection of CPXV particles obtained from clinical samples.

## Materials and Methods

### Ethics statement

All animal experiments were registered and approved by the responsible governmental authorities (Office for Health and Social Affairs Berlin, LaGeSo; registration number H 0349/08). Animals were housed according to national regulations. The physical condition of the animals was monitored daily. No animal became severely ill or died at any time prior to the experimental endpoint. Mice were euthanized by cervical dislocation. Every endeavor was made to minimize pain and distress during the production of poly- or monoclonal antibodies following established best practices [28].

### Proteins and antibodies

The following reagents were obtained through the NIH Biodefense and Emerging Infections Research Resources Repository, NIAID, NIH: vaccinia virus (WR) rA27 (NR-2622) and L1Δ (NR-2625) proteins with a C-terminal histidine tag, recombinantly expressed from baculovirus, as well as human polyclonal anti-VACV immune globulin G (VIG, NR-2632). If not stated otherwise, *E*. *coli*-derived rA27 and L1Δ (see below for production and purification) were used, while the usage of rA27 and L1Δ from the NIH Biodefense and Emerging Infections Research Repository is noted as BEI Resources. A polyclonal rabbit anti-VACV_LE_ antibody was obtained from Acris Antibodies (Herford, Germany). Streptavidin peroxidase (SA-POD), horseradish peroxidase (HRP) labelled donkey anti-goat IgG (H+L), goat anti-mouse IgG (Fc-γ) and goat anti-rabbit IgG (H+L) antibodies or fluorescein (FITC) labelled goat anti-rabbit IgG (H+L) and rabbit anti-goat IgG (H+L) antibodies were obtained from Dianova (Hamburg, Germany), while HRP labelled goat anti-human IgG (Fc-γ) was obtained from Life Technologies (Darmstadt, Germany). Streptavidin-PolyHRP40 was from Diavita (Heidelberg, Germany).

### Plasmids, bacterial strains, viruses and cell lines

Primers were purchased from TIB MOLBIOL (Berlin, Germany). Restriction enzymes, the pTriEx-3 plasmid and the Rosetta™ strain of *E*. *coli* used for recombinant expression of A27 were obtained from Life Technologies (Darmstadt, Germany), while L1Δ, D8Δ and H3Δ were cloned into pQE100 S vectors (Qiagen, Hilden, Germany) and expressed in BL21 DE3 *E*. *coli* strains (Agilent Technologies, Böblingen, Germany).

The VACV strains New York City Board of Health (NYCBOH, VR-1536™) and IHD-W (VR-1441™), HEp-2 cells (CCL-23™) and Vero E6/7 cells (CCL-81™) as well as Tanapox virus (Yaba-like disease virus; VR-937™) were obtained from the American Type Culture Collection (ATCC, Manassas, VA, USA). The VACV strains Lister Elstree (LE) and Modified Vaccinia Ankara (MVA) were obtained from Bavarian Nordic (Martinsried, Germany). The CPXV strains used in this study were isolated from New World monkeys (CPXV_calpox_) [29] or pet rats (CPXV_Kre_) [30]. Camelpox virus (CMLV) strain CP-19 [31], ECTV strains Nü-1 [32] and Moscow, as well as MPXV strain MSF6 [33] were kindly provided by Hermann Meyer (Bundeswehr Institute of Microbiology, Munich, Germany). Parapoxvirus (PPV) ovis strains D-1701 [34] and ORF were kindly provided by Achim Rziha (Friedrich-Loeffler-Institute, Tuebingen, Germany). Herpes Simplex Virus-1 was isolated in our lab from a patient.

### Virus propagation and quantification

The production and titration of viral stocks was done as described previously [35]. All OPV strains were propagated on HEp-2 cells, except for VACV strain MVA, which was propagated on primary chicken fibroblasts isolated from incubated eggs. Vero E6/7 cells were used to propagate PPV and HSV-1 and to determine infectious titers, either as plaque forming units (PFU) or as tissue culture infective dose (TCID_50_ for HSV-1) using the Spearman Kaerber method [36]. Viruses were used as clarified cell culture supernatants or further purified by ultracentrifugation through a 40% sucrose cushion as previously described [37]. For UV inactivation, purified virus particles (adjusted to 1 × 10^9^ PFU/mL in PBS containing 10 μg/mL trioxsalen [Calbiochem, Darmstadt, Germany]) were incubated for 10 min at RT and 10 min UV-irradiated with 1.4 J/cm^2^ (Stratalinker 2400; Stratagene, CA, USA). If not explicitly stated otherwise, all viral strains were tested in native state to ensure the detection of live virus and to exclude exclusive reactivity against inactivated virus. UV inactivated virus was used in the indirect ELISA during initial screenings for mAb generation for safety and technical reasons.

### Expression and purification of A27, D8Δ, H3Δ and L1Δ in *E*. *coli*

Full length CPXV_calpox_ derived recombinant A27 (rA27) was produced as described previously [38]. Extracellular domains of VACV_NYCBOH_ derived D8Δ (aa 2–260), H3Δ (aa 21–270) and L1Δ (aa 1–175) were custom-made by Genexpress (Berlin, Germany). Briefly, the following primers and restriction enzymes (restriction sites underlined) were used for cloning: L1ΔF (*Nde*I) CGTCGGCATATGGGTGCCGCGGCAAG, L1ΔR (*Nsi*I) CCTGTACATGCATTTGTTTAGGTGCTATTT, D8ΔF (*Nde*I) CGTCGGCATATGCCGCAACAACTATCTCCT, D8ΔR (*Nsi*I) CCGACGATGCATCTCTCTCAAATCGGACAACCATC, H3ΔF (*Nde*I) CGTCGGCATATGACATTTCCTAATGTTCAT and H3ΔR (*Bam*HI) CGTCGGGGATCCTTATCCTGGATAACGTTTAG. Expression was induced with 2 mM isopropyl β-D-1-thiogalactopyranoside for 3 h at 37°C, His-tagged proteins were isolated under native (D8Δ) or denaturing (all other proteins) conditions and purified using Protino® Ni-IDA columns (Macherey-Nagel, Dueren, Germany) according to standard procedures. *E*. *coli* lysate was produced as previously described [39].

### Generation of pAbs

PAbs against rA27, D8Δ and H3Δ were generated in goats using TiterMax® Gold (Sigma-Aldrich, Taufkirchen, Germany) adjuvant at a 1:1 ratio. Goats were immunized subcutaneously at two sites and boosted once before serum was collected two weeks later. For rA27, 200 μg of antigen was applied for the primary immunization and 300 μg for the booster immunization four months later; 250 μg (D8Δ) or 150 μg (H3Δ) of antigen was used for the primary and booster immunizations three weeks later, respectively. PAbs against L1Δ were custom-made in rabbits by Open Biosystems (Thermo Fisher Scientific, Dreieich, Germany) using the standard 70-day protocol as described by the manufacturer. IgG was purified from polyclonal sera by protein G affinity chromatography using standard procedures.

### Generation of anti-A27 mAbs

Two 14-week-old female BALB/c and two C57BL/6 mice (BfR Marienfelde, Berlin, Germany) were immunized subcutaneously with TiterMax® Gold as the adjuvant. Mice were primed with 40 μg rA27 (1:1 adjuvant) and boosted 4 weeks later (80 μg rA27; 2:1 adjuvant). Two weeks after the boost, mice were bled; sera were tested for binding to rA27 and the *E*. *coli* lysate. The mouse with the highest titer against rA27 and the lowest cross reactivity to the *E*. *coli* lysate was selected for fusion. After a third subcutaneous boost with 110 μg recombinant protein without adjuvant two weeks later, the selected mouse (C57BL/6) received three final intraperitoneal boosts on days –3, –2 and –1 (110 μg protein each w/o adjuvant) prior to fusion. Hybridoma cells were produced by the fusion of spleen cells with the myeloma cell line P3-X63-Ag8.653 (ATCC), as described previously [40].

Starting from day 10 post-fusion, hybridoma supernatants were screened by a multistep approach to find specific, antigen capture ELISA-suited, antibodies. Indirect ELISAs against rA27 or L1Δ, *E*. *coli* lysate and UV-inactivated VACV_NYCBOH_ were performed by coating 200 ng protein or 5 × 10^5^ PFU virus per well and incubated with 50 μL hybridoma supernatant. Bound mouse antibodies were detected by HRP-labelled goat anti-mouse IgG (Fc-γ). Antigen capture ELISA was done by coating 200 ng/well polyclonal rabbit anti VACV_LE_, capturing 5 × 10^5^ PFU/well native VACV_NYCBOH_ and detection using 50 μL hybridoma supernatant followed by the goat anti-mouse (Fc-γ) specific HRP-labelled detection antibody. The procedures for both ELISAs are described below.

Hybridoma cells were sub-cloned at least once by limiting dilution and tested for stable and monoclonal intracellular IgG production by flow cytometry [41]. IgG was isolated from hybridoma culture supernatants by affinity chromatography on HiTrap Protein G HP columns and an ÄKTA Explorer purification platform (GE Healthcare, Freiburg, Germany) according to the manufacturer’s instructions. The IgG concentration was determined by absorption measurements using a Nanodrop 1000 (Thermo Fisher Scientific). Antibodies were isotyped using the Pierce Rapid ELISA Mouse mAb Isotyping Kit (Thermo Fisher Scientific).

### Coupling of antibodies to biotin and DyLight649

Protein G purified antibodies (1 mg/mL in PBS pH 7.3) were coupled with Sulfo-NHS-LC biotin (Thermo Fisher Scientific) at a 20-fold molar excess of biotin according to standard procedures. Fluorochrome coupling was performed at a 15-fold molar excess of DyLight 649 NHS Ester (Thermo Fisher Scientific) leading to molar coupling ratios between 2 and 8 DyLight molecules per IgG molecule (measured and calculated as described by the manufacturer).

### ELISA

Indirect ELISA for testing antigens or pAbs was performed as described previously [38]. Recombinant proteins were coated at a concentration of 200 ng/well, and viral particles (UV-inactivated VACV_NYCBOH_) were adjusted to 5 × 10^5^ PFU/well.

For antigen capture ELISA, 200 ng capture antibody was coated overnight at 4°C in 100 μL coating buffer (0.1 M NaHCO_3_, pH 9.6) to MaxiSorp™ ELISA plates (Nunc, Langenselbold, Germany). Plates were washed four times with 300 μL Tris-buffered saline (0.1 M Tris, 0.9% (w/v) NaCl, pH 7.5) containing 0.05% Tween 20 (TBS-T) between all incubation steps. After coating, plates were blocked for 2 h at RT with 300 μL TBS-T containing 3% bovine serum albumin (BSA, Carl Roth, Karlsruhe, Germany). Subsequently, 100 μL viral particles or rA27 diluted in TBS-T containing 0.25% BSA were incubated for 1 h at 37°C. After washing, 100 μL biotinylated detection antibody (200 ng/mL diluted in TBS-T + 0.25% BSA) were incubated for 1 h at 37°C. Finally, 100 μL HRP-coupled streptavidin was added at 200 ng/mL for 30 min at 37°C. Detection was achieved by incubating 100 μL 3,3’,5,5’-tetramethylbenzidine (TMB; Sigma-Aldrich) substrate solution for 15 to 30 min before stopping the enzymatic reaction by adding 100 μL 2 M H_2_SO_4_. Absorbance was read at 450 nm and referenced to 620 nm using an Infinite200 PRO microplate reader (Tecan, Maennedorf, Switzerland).

For final testing of the detection of different OPV strains from clarified cell culture supernatants as well as detection in clinical samples, the antigen capture ELISA protocol was further optimized to include a polymeric horseradish peroxidase coupled to streptavidin (SA-polyHRP40) for enhanced sensitivity as described elsewhere [40], with the exception that the capture antibody A1/40 was coated at 5 μg/mL instead of 10 μg/mL.

### Western blot analysis

For western blot analysis, HEp-2 cells were infected with VACV_NYCBOH_ at an MOI of 0.2 and cultivated for 3 to 4 days. Cells were lysed using RIPA lysis buffer supplemented with the HALT Protease Inhibitor cocktail (Thermo Fisher Scientific) and protein concentrations were determined using a BCA protein assay kit (Thermo Fisher Scientific). A protein lysate from mock-infected HEp-2 cells was used as the negative control. Proteins were separated on 8 to 16% precast gradient PAA gels (Pierce Precise™ Protein Gels, Thermo Fisher Scientific) and blotted onto a 0.2 μm PVDF membrane (VWR, Darmstadt, Germany). A PageRuler™ pre-stained standard (Fermentas, St. Leon-Rot, Germany) was used as the molecular weight marker. Membranes were blocked in TBS-T supplemented with 5% skim milk (Carl Roth) at 4°C overnight. Primary antibodies were incubated for 1 h at RT diluted in TBS-T supplemented with 1% skimmed milk, as were HRP-labelled species-specific secondary antibodies. Detection was done via chemiluminescence after 5 min incubation with ECL western blotting substrate (Thermo Fisher Scientific).

### Immunofluorescence assay (IFA)

Glass slides (12 well; Menzel-Glaeser, Braunschweig, Germany) covered with VACV_LE_ infected HEp-2 cells were fixed in ice cold acetone and stored at –20°C until further use. For immunofluorescence staining, 1:100 dilutions of rabbit anti-VACV_LE_ antibody or goat anti-A27 antibody in PBS (pH 7.3) supplemented with 2% BSA were incubated for 1 h at 37°C in a humid chamber. After three washing steps with PBS, DyLight 649 labelled antibodies (1:100) were incubated simultaneously with 1:500 dilutions of either FITC-labelled goat anti-rabbit IgG (H+L) or rabbit anti-goat IgG (H+L) for 1 h at 37°C. Cell nuclei were counterstained by adding 20 μL of a 1.4 μM solution of 4',6-diamidino-2-phenylindole (DAPI; Invitrogen) for 10 min at 37°C. Slides were mounted with fluorescence mounting medium (Dako, Glostrup, Denmark) and examined using an LSM 510 META confocal laser scanning microscope (Carl Zeiss, Oberkochen, Germany).

### Plaque reduction neutralization test (PRNT)

Antibody-mediated neutralization was determined according to Newman et al. [42]. The antibody concentration which resulted in a 50% plaque reduction (PRNT_50_) was calculated by the Reed-Muench/Spearman Kaerber method [36].

### Electron microscopy

Immuno-negative staining and electron microscopy were performed as described elsewhere (Laue, 2010). Briefly, purified VACV_NYCBOH_ particles were inactivated by incubation in freshly prepared 2% PFA in 0.05 M HEPES (pH 7.2), sonicated and immobilized on sample supports for transmission electron microscopy. Biotinylated pAbs were titrated on BSA coated grids, until detection with 5 nm gold nanoparticle coupled streptavidin (British Biocell, Cardiff, United Kingdom) resulted in the same mean background labelling density of ~10 particles per view field at a, 87,000-fold magnification (anti-A27: 0.7 μg/mL; anti-D8: 2 μg/mL; anti-H3: 0.9 μg/mL; anti-L1: 1.9 μg/mL). Negative staining was performed with either 0.1 or 0.5% uranyl acetate solution. For quantification, only IMV particles of the mulberry form, which were found isolated from other particles [43], were analyzed. Randomized sampling was done in 22 evenly distributed mesh areas with five viral particles analyzed per area. Imaging was done with a Tecnai 12 BioTwin (FEI Corp.) at 120 kV and a 1k digital CCD camera (Megaview III, Olympus Soft Imaging Solutions). Raw data can be accessed under https://zenodo.org/record/45197 (DOI: 10.5281/zenodo.45197).

### Epitope mapping

A peptide microarray spotted with synthetic 15mers with an overlap of 11 amino acids (JPT, Berlin, Germany) covering the full-length A27 sequence of VACV WR (UniProtKB: P11258) was used for epitope mapping. Spots of rA27 protein (BEI Resources) were included as positive controls, and a random 15mer peptide was included as a negative control. MAbs diluted to 10 μg/mL in TBS were incubated on glass slides for 2 h at 37°C. After washing, DyLight 649 coupled goat anti-mouse IgG (H+L) (Thermo Fisher Scientific; 1 μg/mL) was added for 1 h. The slides were analyzed with a GenePix 4000B microarray scanner (Molecular Devices, Bieberach an der Riss, Germany) and analyzed using GenePix Pro software version 6.1.0.4.

### SPR measurements

Kinetic analysis was carried out at 25°C using a Biacore T100 SPR system (GE Healthcare). L1Δ and rA27 (both BEI Resources) were coupled covalently to CM5 sensor chips (GE Healthcare) using standard amine-coupling chemistry to surface densities below 62 resonance units (RU) to avoid mass transport limitation during kinetic measurements. For the determination of binding kinetics, mAbs were two-fold serially diluted in HSB-N buffer (10 mM HEPES, 150 mM NaCl, pH 7.4) ranging from 16 μg/mL (107 nM) to 0.5 μg/mL (3.33 nM) with duplicate measurements at the highest concentration. All measurements were carried out at a flow rate of 30 μL/min. The association phase was monitored for 120 s followed by buffer injections for 420 s (900 s for highly stable antibodies) to measure the dissociation phase. Binding responses were normalized by subtracting non-specific binding to flow cell 1 (L1) from flow cell 2 (A27). The sensor surface was regenerated by a 30 s injection of 10 mM glycine-HCl buffer (pH 1.5). For the determination of the kinetic binding parameters, double referenced [44] binding curves were fitted to a 1:1 Langmuir binding model with the maximum binding capacity R_max_ fitted locally. The measured association rate constants *k*_a_, dissociation rate constants *k*_d_, equilibrium binding affinities *K*_D_, and R_max_ were determined in three independent experiments.

## Results

### Characterization of antibodies against recombinant surface proteins A27, D8, H3 and L1

The aim of this work was to develop an antigen capture ELISA optimized for the detection of OPV. Often, antibodies implemented in immunological assays are generated against whole viral particles. However, which individual protein of the viral surface is targeted by an antibody might influence the assay performance. Until now, no systematic evaluation as to which protein enables efficient antibody binding for subsequent viral particle detection has been performed.

**Fig 1:**
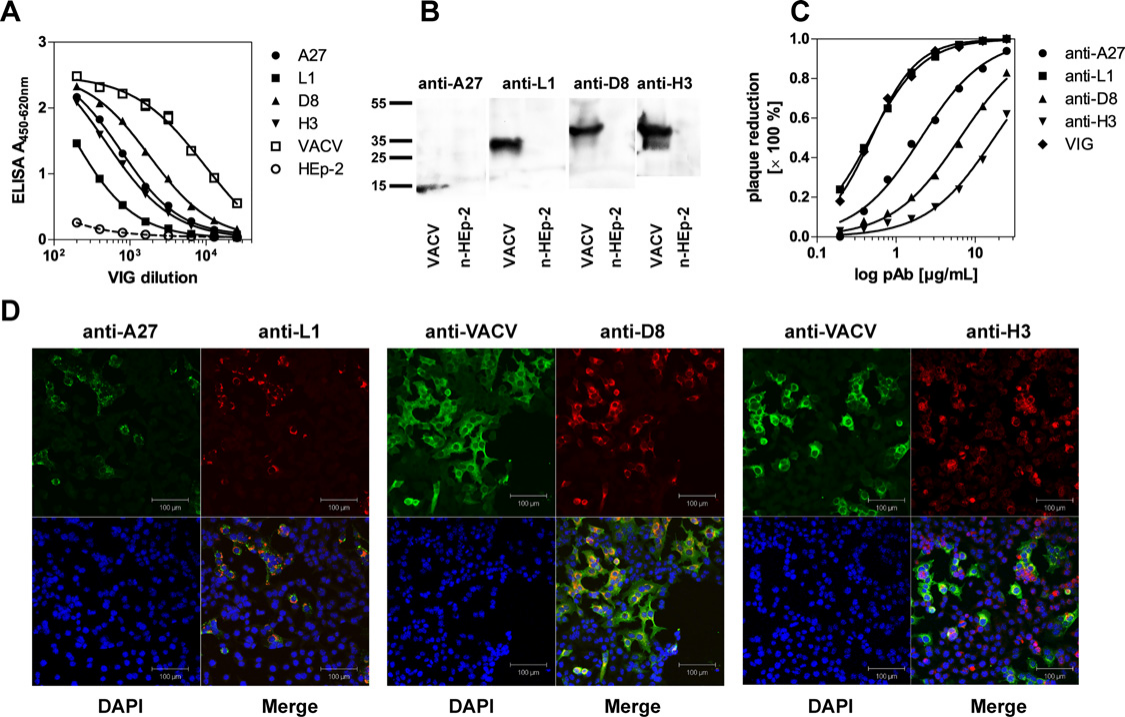
Characterization of polyclonal antibodies against recombinant surface proteins A27, L1, D8 and H3. **A**. Conservation of immunogenic epitopes of recombinant proteins was verified through the recognition of immobilized surface proteins by vaccinia immunoglobulin (VIG) employing an indirect ELISA. Lysates from vaccinia virus infected (VACV_NYCBOH_) or non-infected HEp-2 cells were used as the positive or negative control. **B**. The reactivity of anti-A27, -L1, -D8 and -H3 antibodies (1:1000) against VACV-infected (strain NYCBOH) or non-infected (n-) HEp-2 cells (40 μg lysate per lane) by western immunoblotting. HRP-labelled goat anti-rabbit or rabbit anti-goat IgG antibodies (1:5000) were used for detection. **C**. Percentage of plaque reduction (PRNT; ranging from 0% to 100%) for two-fold serial dilutions of anti-A27, -L1, -D8 and -H3 antibodies. VIG was included as the positive control. **D**. IFA of VACV_LE_ infected Hep-2 cells with different combinations of polyclonal antibodies targeting surface proteins (A27, L1, D8 and H3) or vaccinia virus (VACV). Compared to the other antibodies, for the analysis of anti-H3 stained infected cells, the detector gain had to be increased due to the lower signal intensity. Antibodies were either stained with species-specific FITC-labelled secondary antibodies (green) or labeled directly with DyLight 649 (red). Cell nuclei were counterstained with DAPI (blue). Scale bar = 100 μm.

To address this question, pAbs targeting four recombinant viral proteins (A27, L1, D8 and H3) were generated in this work. Before immunization, sustained binding of VIG to the recombinant proteins (Fig 1A) demonstrated the preservation of immunogenic epitopes in the proteins.

Subsequently, purified pAbs were characterized using different assays to ensure that potential differences in binding to viral particles were physiologically relevant and not skewed by testing antibodies against misfolded recombinant proteins.

First, the antibodies were tested for specific recognition of their respective viral target in a VACV-infected HEp-2 cell lysate by immunoblotting (Fig 1B). Here, all antibodies showed exclusively immunoreactivity with proteins at the expected molecular weight for A27 (12.6 kDa), L1 (27.3 kDa), D8 (35.5 kDa) and H3 (37.5 kDa), whereas no binding to non-infected HEp-2 cells was observed.

Next, the ability of the generated pAbs to neutralize VACV_NYCBOH_ infections of Vero E6/7 cells was tested by PRNT (Fig 1C). As previously described, antibodies recognizing the OPV surface proteins used in this work have all been shown to neutralize virus entry into host cells [45–52]; this test was included to test for functional virus binding. Here, anti-L1 antibodies inhibited virus infection most efficiently (PRNT_50_: 0.47 μg/mL) comparable to virus neutralization by VIG, which was used as the positive control (PRNT_50_: 0.49 μg/mL). Anti-A27 (PRNT_50_: 2.1 μg/mL), anti-D8 (PRNT_50_: 6.4 μg/mL) and anti-H3 antibodies (PRNT_50_: 17.5 μg/mL) also inhibited viral infection, though to a lesser extent.

Finally, the pAbs were tested by IFA with VACV_LE_-infected HEp-2 (Fig 1D). Here, co-localization of anti-A27 and anti-L1 antibodies within infected cells and co-localization of anti-D8 antibodies with a polyclonal rabbit anti-VACV antibody indicated specificity for virus-infected cells as well as the ability of the antibodies to recognize the native viral antigen in its cellular context. Conversely, the fluorescence intensity of anti-H3 antibodies was significantly lower compared to the other tested antibodies. Therefore, the detector gain was increased to achieve similar signal intensities compared to the other antibodies tested, which also led to higher background signals from non-infected cells.

In conclusion, anti-A27, -D8 and -L1 pAbs generated by immunization with recombinant surface proteins reacted with native viral epitopes as shown by virus neutralization and specific staining by IFA. In contrast, anti-H3 pAbs exhibited poor virus neutralizing capacities and weak staining of infected cells by IFA, indicating that this antibody did not recognize native viral epitopes.

### Binding properties of anti-A27, -D8, -H3 and -L1 antibodies to virus particles

Next, it was tested if differences in the accessibility and overall amount of viral surface proteins had an influence on the binding of antibodies to viral particles. To this aim, the binding of the surface protein-specific antibodies to purified virus particles was tested by two approaches.

**Fig 2:**
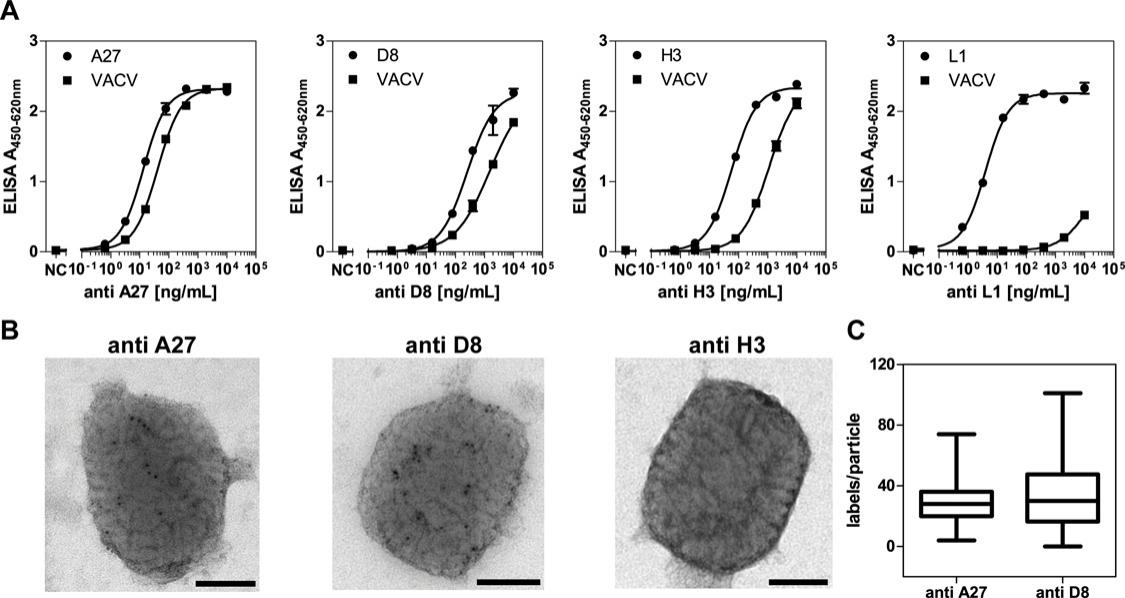
Binding to viral particles by anti-A27, -D8, -H3 and -L1 pAbs. **A**. Immobilized recombinant proteins or purified UV-inactivated VACV_NYCBOH_ particles were incubated with a dilution series of purified biotinylated anti-A27, -D8, -H3 and -L1 antibodies in an indirect ELISA. Detection was done with SA-pHRP (1:5000). **B**. Representative electron microscopic pictures of anti-A27, -D8, or -H3 antibodies binding to purified VACV_NYCBOH_. Biotinylated antibodies were detected using 5 nm immunogold labelled streptavidin. Scale bars = 100 nm. **C**. Box-and-whisker plot for quantification of the number of gold particles per viral particle (whiskers: min to max; A27 n = 111; D8 n = 109 viral particles analyzed).

First, binding of pAbs to immobilized VACV particles was compared by indirect ELISA (Fig 2A). Differences in antibody titers were taken into account by calculating the EC_50_-ratio of binding to VACV by binding to the recombinant proteins. Here, both anti-A27 and anti-D8 antibodies bound immobilized viral particles similarly well (EC_50_-ratios: A27 37%; D8 17%), while anti-H3 antibodies showed less binding (EC_50_-ratio: 5%). Interestingly, despite the high neutralizing capacity of the anti-L1 antibody, virus binding was the lowest (EC_50_-ratio: 0.04%).

Second, to test if these differences were caused by the number of accessible protein molecules on the viral surface, antibody binding to purified VACV was studied by immune-negative staining transmission electron microscopy. Here, both anti-A27 and anti-D8 antibodies (biotinylated and visualized by streptavidin coupled to Au nanoparticles) labelled VACV particles to a similar degree, while no labelling could be seen for anti-H3 (Fig 2B) or anti-L1 (data not shown) antibodies at the tested antibody concentrations. When quantified, the median number of gold labels per virus particle (n) did not differ significantly between A27 and D8 (A27 n = 28; D8 n = 30; Mann-Whitney U test p = 0.2869), though the variability for anti-D8 antibodies was higher (Fig 2C). Moreover, while gold particles after anti-A27 staining were easily visible after standard negative staining (0.5% uranyl acetate), a similar visibility of gold particles after anti-D8 staining required negative staining with a reduced uranyl acetate concentration (i.e. 0.1%). This difference in the visibility of the gold label could be related to a principal difference in the localization of the respective epitopes (e.g. D8 epitopes could be localized in grooves rather than on the flat surface of the virus particle or in a protein-rich surrounding, both of which would be filled with uranyl acetate during staining, thereby interfering with the detection of gold particles by reducing the contrast).

Based on these results, A27 was chosen for the subsequent generation of mAbs, though D8 might also be a promising target to establish detection-optimized antibodies.

### Generation of antigen capture ELISA-suited anti-A27 mAbs

To assure specificity for OPV and to exclude antibodies against cellular contaminants or the His-tag of rA27, a multistep screening approach was applied. After immunization and fusion of mouse spleen cells with myeloma cells, primary screening was performed by indirect ELISA against rA27 versus the *E*. *coli* lysate. Only hybridoma cells producing antibodies with high reactivity against rA27 and low reactivity against the *E*. *coli* lysate were transferred to new 96-well plates for re-screening. In the re-screening, binding to rA27 versus binding to His-tagged L1 was assessed to check for sustained antibody production and to exclude recognition of the His-tag. Finally, to assure recognition of the native conformation, binding to VACV was tested by indirect and antigen capture ELISA.

The screening system was highly efficient in narrowing down the tested hybridoma clones to highly specific producers of antibodies suitable for antigen capture ELISA (S1 Fig). Although the majority of tested hybridoma cells (*n* = 2288) showed reactivity against rA27 (73% with A27 ELISA OD > 1.0 while *E*. *coli* OD < 1.0), only eight clones produced antibodies able to detect VACV by antigen capture ELISA.

### Characterization of anti-A27 mAbs

Subsequently, purified mAbs were isotyped, tested for the detection of VACV-infected Hep-2 cells by IFA and assayed for virus neutralization by PRNT. All eight antibodies specifically stained VACV-infected cells (S2 Fig). However, only four antibodies exhibited some virus neutralization, while the others did not reduce plaque numbers even at the highest antibody concentration tested (Table 1).

**Table 1:** Characterization of anti-A27 mAbs by PRNT, epitope mapping and SPR measurements.

Antibody	Isotype	PRNT_50_ [μg/mL]	*k*_a_ [10^5^ M^-1^s^-1^]^a^	*k*_d_ [10^−4^ s^-1^]^a^	*K*_D_ [10^−9^ M]^a^	R_max_ [%]^a^^,^^b^
A1/6	IgG1	22	2.5 ±0.1	5 ±2	2.0 ±0.8	78 ±16
**A1/40**	**IgG1**	**19**	2.3 ±0.3	5 ±2	2.5 ±0.9	36 ±5
A1/77	IgG2b	> 25	0.64 ±0.04	1.4 ±0.4	2.3 ±0.6	31 ±2
A1/116	IgG2b	> 25	1.5 ±0.1	1.7 ±0.5	1.1 ±0.4	81 ±16
A2/320	IgG2b	> 25	0.95 ±0.06	0.8 ±0.3	0.8 ±0.3	59 ±10
A2/654	IgG2b	19	0.96 ±0.02	0.8 ±0.2	0.8 ±0.2	60 ±10
A3/509	IgG2b	> 25	1.8 ±0.3	2.0 ±0.5	1.1 ±0.3	100 ±1
**A3/710**	**IgG2b**	**11**	4.3 ±0.03	8 ±1	1.7 ±0.3	33 ±4

^a^ Mean ± standard deviation of 3 independent experiments.

^b^ Normalized to highest R_max_ measured in each independent experiment (A3/509). As surface densities of A27 were varied between experiments, comparison of absolute values is not possible.

**Fig 3:**
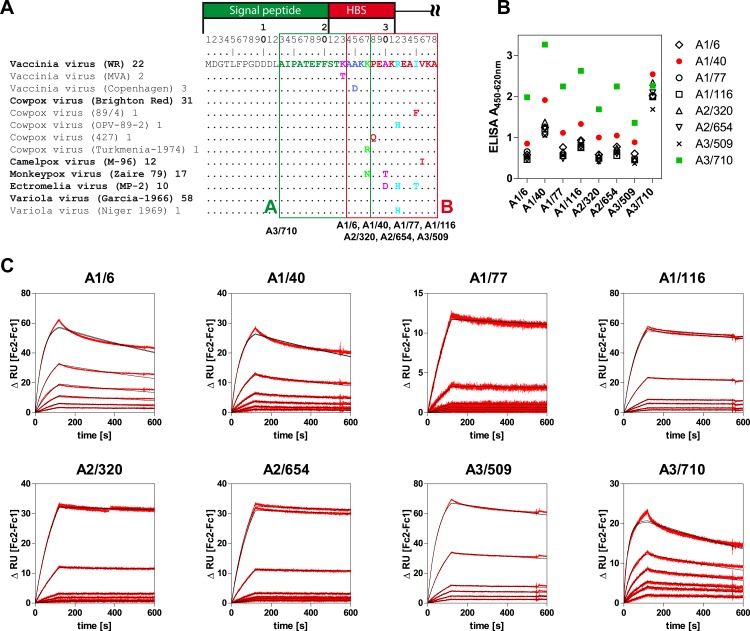
Characterization of monoclonal anti-A27 antibodies. **A**. Binding epitopes and multiple sequence alignment (NCBI BLink) of different OPV strains. Identical sequences with the prominent strain are denoted in brackets, followed by the number of identical sequences deposited in GenBank. The binding epitopes for mAb A3/710 from amino acid 13 to 27 (A) and all other anti-A27 mAbs from amino acid 24 to 38 (B) border the heparin binding site (HBS) on A27. Both the VACV_NYCBOH_ strain and *E*. *coli* derived A27 based on CPXV_calpox_ DNA show 100% sequence identity with the reference strain VACV_WR_ in this region. **B**. Compatibility of all eight anti-A27 mAbs for antigen capture ELISA. All possible combinations of coating antibodies (x-axis) and biotinylated detection were paired and tested for the detection of 5×10^6^ PFU/ml UV-inactivated VACV_NYCBOH_. Shown are representative results for the highest virus concentration tested. The results from the titration series are shown in S4 Fig. **C**. SPR sensorgrams to determine the binding kinetics of anti-A27 mAbs. Measured responses (double referenced resonance units RU; difference between flow cell 2 minus flow cell 1) are shown as red lines whereas black lines represent results from fitting a 1:1 Langmuir interaction.

The exact binding epitopes within A27 were determined by peptide epitope mapping (Fig 3A and S3 Fig). Here, mAb A3/710 bound to a peptide ranging from aa 13 to 27 (epitope A), while the remaining seven anti-A27 mAbs bound to a peptide comprising aa 24 to 38 (epitope B). As the signal peptide (aa 1 to 20) is cleaved during viral maturation [53] and A3/710 recognized infected cells, the epitope of A3/710 can be further narrowed to the outermost N-terminal amino acid sequence STKAAKK of mature A27. Except for single mutations found in only very few strains, both epitopes are highly conserved on the species level between VACV, CPXV and VARV. In contrast, both ECTV (A30D, R32H, I35T) and MPXV (K27N, A30T) exhibit mutations, mainly in epitope B, which could potentially interfere with detection of these OPV species.

To test which of the anti-A27 mAbs could be combined in an antigen capture ELISA, all capture/detection-antibody combinations were tested and compared by their ability to detect VACV_NYCBOH_ (Fig 3B). Here, combinations of mAb A1/40 and mAb A3/710 were clearly superior to the other combinations tested. A3/710 mAb showed superior assay performance with all other mAbs tested, regardless of being used as a capture or detection antibody, matching the fact that it recognizes a different epitope.

Finally, the association rate constants (*k*_a_), dissociation rate constants (*k*_d_) and equilibrium dissociation constants (*K*_D_) for all mAbs were determined by SPR measurements (Fig 3C and Table 1). Here, all mAbs bound to immobilized A27 with high sensitivity resulting in affinity constants K_D_ between 0.8 nM and 2.5 nM. Despite the fact that seven mAbs recognized the same epitope, different binding modes emerged with respect to *k*_a_, *k*_d_ and R_max_. The kinetic interaction of the mAbs was either characterized by rapid but less stable binding as exemplified by mAbs A1/6 and A1/40, or by a slightly slower but also more stable binding as exemplified by mAbs A2/320 and A2/654. Additionally, the maximum binding capacity R_max_, which can be used as a measure of the stoichiometry of an interaction, differed between the tested mAbs. Here, three groups, characterized by high (A1/6, A1/116, A3/509), medium (A2/320, A2/654), or low (A1/40, A1/77, A3/710) maximum binding capacity could be discerned.

### Establishment and pre-validation of an anti-A27 antigen capture ELISA

**Fig 4:**
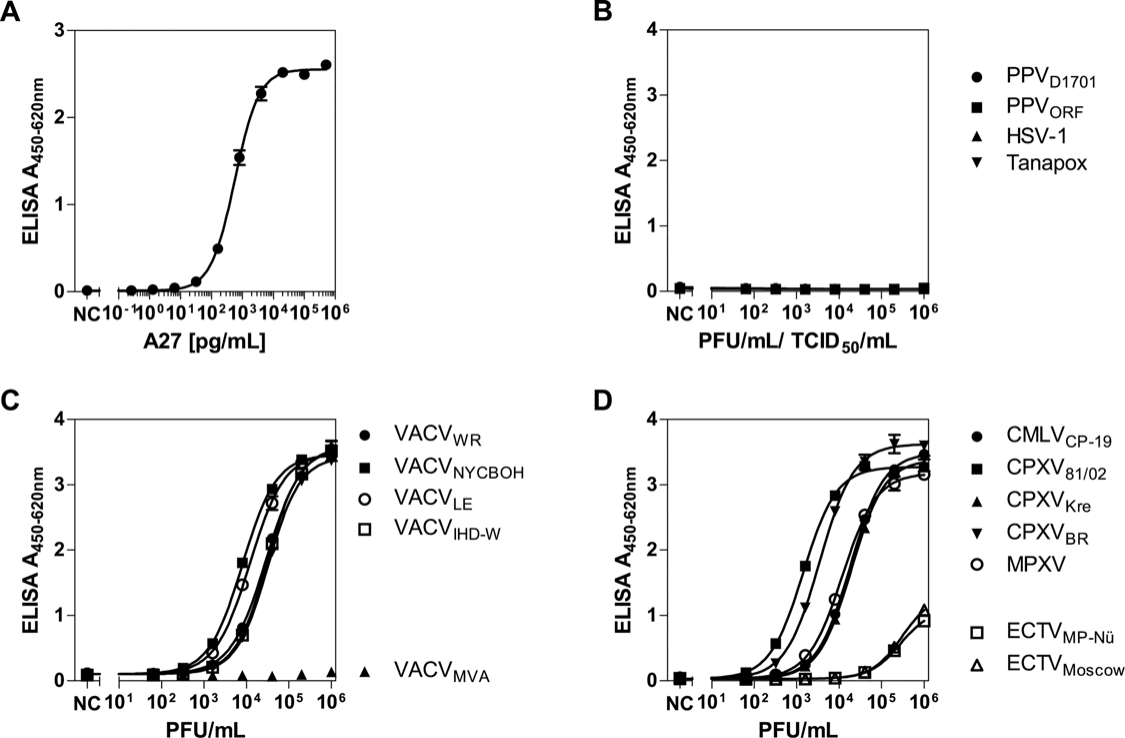
Antigen titration curves for the newly developed antigen capture ELISA with mAb A1/40 as capture and mAb A3/710 as detection antibody. **A**. Titration curve of rA27 with the antigen capture ELISA. **B**. To check for cross-reactivity, PPXV, HSV-1 and tanapox virus were tested. **C**. Detection of different VACV strains by the newly developed assay. **D**. Detection of different OPV.

Based on these results, an antigen capture ELISA with mAb A1/40 as the capture antibody and mAb A3/710 as the detection antibody was established. The novel ELISA allowed for highly sensitive detection of rA27 with a limit of detection of 5 pg/mL (95% CI: 4–7 pg/mL; Fig 4A). No cross-reactivity was observed against PPV, HSV-1 and Tanapox virus (Fig 4B). All tested VACV strains except VACV_MVA_, which was not detectable, could be detected with high sensitivity (Fig 4C). CMLV, CPXV and, most importantly, MPXV were also detected at limits of detection between 1.4 × 10^1^ and 2.9 × 10^2^ PFU/mL. ECTV could also be detected, though at lower sensitivity (Fig 4D and Table 2).

**Table 2:** Limits of detection (LOD) of the newly developed A27-specific antigen sandwich ELISA for different OPV strains.

Virus Strain	LOD (95% CI) [PFU/mL]^a^
VACV_WR_	4.3 × 10^2^ (3.0–6.7)
VACV_NYCBOH_	1.5 × 10^2^ (1.3–1.9)
VACV_LE_	2.0 × 10^2^ (1.6–2.6)
VACV_IHD-W_	6.8 × 10^2^ (5.1–9.6)
VACV_MVA_	negative
CPXV_81/02_	1.4 × 10^1^ (1.1–2.1)
CPXV_Kre_	2.0 × 10^2^ (1.5–3.1)
CPXV_BR_	2.9 × 10^2^ (1.9–5.4)
CMLV_CP-19_	1.7 × 10^2^ (1.4–2.1)
ECTV_MP-Nü_	1.1 × 10^4^ (0.6–2.5)
ECTV_Moscow_	1.0 × 10^4^ (0.9–1.2)
MPXV_MSF6_	1.1 × 10^2^ (0.8–1.8)

^a^ Cutoff = mean of blank + 3.29 standard deviations. Duplicate measurements were made. The 95% confidence interval (CI) as calculated by fitting a four-parametric Hill-slope curve to log-transformed virus concentrations.

Finally, a panel of 11 CPXV-positive samples (confirmed by qPCR as described by Nitsche et al., 2004) isolated from different species (human, rat, cat, horse and cheetah) and including different kinds of sample material (swabs from lesions, homogenized biopsy material from lung, crust, hair and skin) was re-evaluated using the newly established antigen capture ELISA (S5 Fig and Table 3). To account for potential unspecific binding causing false positive results, samples were also incubated with a negative control antibody (anti-ricin mAb R109 described by Pauly et al., 2009) which was of the same IgG1 isotype as mAb A1/40 used for specific detection. Here, seven out of ten samples (tested at a 1:10 dilution) were identified as OPV-positive by ELISA, whereas three qPCR positive samples but with a very low viral DNA load (C_T_-values > 30) tested negative.

**Table 3:** Detection of CPXV from clinical samples.

Sample	qPCR [C_t_]	ELISA Results^a^
Human Swab	21.9	++++
Human Swab	18.8	++
Rat Skin	14.2	+
Cheetah Lung	11.9	++++
Human Lung	10.8	+++
Horse Crust	18.1	++++
Cat Crust	15.8	+++
Horse Swab	32.3	-
Cat Hair	34.1	-
Human Crust	35.8	-
CPXV^b^	-	+++

^a^ p-value of unpaired t-test for 1:10 diluted samples between negative control antibody (anti ricin mAb: same IgG1 isotype) and specific capture antibody (A1/40) (n = 2). ++++: p < 0.0001; +++: p < 0.001 ++: p < 0.01; +: p < 0.05; -: negative.

^b^ CPXV_BR_ tested at 1 × 10^4^ PFU/mL as the positive control.

## Discussion

The increased occurrence of zoonotic OPV infections, not least due to an increased susceptibility of today’s population [54], necessitates the development of easily manageable on-site detection methods. Though large panels of mAbs targeting diverse surface proteins of OPV have been described [27, 51, 55–59], their implementation in assays for OPV detection has either not been tested or the tested mAbs were not able to detect all clinically relevant OPV [27]. Here, we describe the development of the first solely mAb-based antigen capture ELISA able to detect all human pathogenic OPV tested with high sensitivity.

### Virus binding properties of surface protein specific antibodies

One goal of this work was to elucidate the relationship between targeting different surface proteins and virus binding. This was done since, despite a tremendous body of work on neutralizing antibodies, the influence of the target protein on the ability to bind viral particles efficiently has not been addressed until now.

To this aim, recombinant proteins instead of live virus particles were used for immunization. This approach bypasses the complex immune response against the multitude of immunogenic viral proteins [38, 39, 60] and instead enables a strong and directed immune response against the target of interest. Four surface proteins were chosen based on previously described induction of neutralizing and protective antibodies [45–52]. In this work, however, binding to viral particles instead of virus neutralization was chosen as the selection criteria.

Here, A27 and D8 were found to be superior to L1 for the induction of antibodies suitable for detection. This was most likely caused by the overall frequency and accessibility of these epitopes on the viral particle surface. Both A27 and D8 are among the most frequent surface proteins, while L1 is found at least ten times less frequently on purified IMV particles [61]. Additionally, both A27 and D8 are presented in a multimeric fashion [8, 52, 62], which enables bivalent interaction of mAbs with the surface proteins, resulting in binding with higher avidity.

The importance of choosing proper targets for viral detection is also underlined by results obtained with single domain antibodies (sdAbs) targeting L1 to establish an antigen capture ELISA for VACV detection [63]. Here, sdAbs selected against L1 failed to detect viral particles in an antigen capture MAGPIX assay despite high affinity for L1. Furthermore, sdAbs selected against viral particles were successful at virus detection in a MAGPIX assay, although at a suboptimal limit of detection of only 4 × 10^5^ PFU/mL. Unfortunately, the protein targets of the latter sdAbs could not be elucidated in detail. However, as shown by the lack of reactivity by indirect ELISA, A27 and L1 could be excluded as targets. Conversely, the newly developed assay established in this work is at least 100 times more sensitive due to careful selection of the optimal protein target and optimal mAbs suited for antigen capture ELISA.

The neutralizing activity of the pAbs was in agreement with previous reports. Here, antibodies targeting L1 mediated potent neutralization [51, 64]. In contrast, anti-A27, -D8 and -H3 antibodies also mediated neutralization [45, 65, 66], but in a complement- [48, 51] or strain-dependent manner. The latter could be explained by the fact that anti-A27, -D8 and -H3 antibodies interfere with virus-GAG interactions, needed for viral entry only by certain virus-cell line combinations [67, 68].

Although all pAbs were neutralizing and able to detect specific bands by western blot, the possibility that the recombinant proteins used for immunization were partially misfolded cannot be ruled out completely, especially in the case of H3. Here, the low signal intensity observed in the IFA indicates that few antibodies were able to detect native H3 on the surface of viral particles. Anti-H3 antibodies were potentially more neutralizing in one study [66]. However, in another study, only moderate neutralization comparable to our results was observed [69]. Nevertheless, the strategy to compare virus-binding properties against several potential targets of detection antibodies augmented by different tests to ensure binding to native viral protein could also be applied to other viral or bacterial targets.

Finally, our results indicate that different modes of antigen presentation can lead to diverging results when polyclonal antibodies are tested in individual assays. When tested by IFA, antibodies can bind to proteins both incorporated in viral particles as well as to freshly translated proteins on crescent viral particles or proteins found loosely in the cytoplasm of the infected cells. Conversely, when binding to purified viral particles is assayed, only epitopes on the surface of densely packed viral particles are accessible for antibody binding. Therefore, the specific detection of infected cells by IFA is a good evidence that the native viral protein can be bound by an antibody. It is, however, not sufficient to ensure binding to native viral particles by the same antibody, which should be tested independently either by ELISA or EM.

### Optimized screening during mAb generation

Our results also demonstrate that choosing a screening method that matches the desired application is of utmost importance to isolate the best-suited antibodies. To this end, highly neutralizing anti-L1 antibodies failed to perform well in binding to viral particles, highlighting that screening for neutralization could bias the population of selected mAbs towards neutralizing but weakly binding antibodies. Moreover, including an antigen capture ELISA in the screening procedure of anti-A27 mAbs was essential for the efficient identification of high-affinity antibodies best suited to be implemented in the same assay format.

### Characterization of anti-A27 mAbs by SPR and epitope mapping

The success of this strategy is also illustrated by the fact that all eight mAbs retrieved by the antigen capture ELISA screening bound A27 with high affinity, as determined by SPR measurements. Here, only two (A2/320, A2/654) of seven mAbs binding to epitope B were identical regarding all parameters determined by SPR (*k*_a_, *k*_d_, K_D_ and R_max_). All other mAbs differed from each other by either binding kinetics, affinity or maximum binding capacity, indicating unique antibodies covering the same epitope with different binding modes.

When the detection of different OPV strains was tested by the newly established antigen capture ELISA, known point mutations in the binding epitopes corresponded to the lack of detection of certain species or isolates. The loss of VACV_MVA_ binding can be explained by the K23T mutation in the epitope recognized by the A3/710 mAb, whereas the lower sensitivity of ECTV detection indicates that one or more of the ECTV-specific mutations (A30D, R32H and I35T) occur at amino acids involved in mAb A1/40 binding. Despite the close proximity of both epitopes on the flexible N-terminal end of A27, the bend introduced by the proline residue at amino acid 28 at the KKPE motif [14] seems to separate both mAb binding sites sufficiently for simultaneous binding. Surprisingly, mAb A1/40 bound to the same peptide epitope as the previously described mAb 5B4, which is neutralizing but unable to bind MPXV as well as ECTV [27, 47]. This indicates that, despite binding to identical epitopes, different amino acids are crucial for the interaction. Conversely, the binding epitope of another antibody highly specific for MPXV A27 was mapped to the same epitope as A1/40, with K27N (MPXV) also being crucial for the interaction [70]. The fact that three mAbs with diverging reactivity bind to the same epitope highlights the flexibility in this A27 region as well as multiple modes of interaction employed by the different mAbs binding to this epitope.

Lastly, our results also show that combining two mAbs with different binding epitopes significantly improves the sensitivity of virus detection. It is well-known that for multimeric proteins with repeating epitopes or viral particles, the same antibody can be used successfully for capturing and detection [71]. Here enough epitopes remained accessible for subsequent detection-antibody binding. Although, in our work, the viruses could also be detected by the use of identical capture and detection antibodies, the sensitivity was much higher when pairs of antibodies binding to different epitopes were combined.

### Assay performance of the newly developed antigen capture ELISA

The newly developed antigen capture ELISA allowed highly sensitive and specific detection of all important human pathogenic OPV tested. As neither the highly attenuated VACV_MVA_, which is not found in the environment, nor ECTV are pathogenic to humans, absent or less sensitive detection for these viruses does not diminish the value of the ELISA for the detection of virulent OPV. The high conservation of both epitopes indicates that VARV as a potential bioterrorist agent is likely to be recognized by both antibodies. The limits of detection were comparable to those of a previously described anti-A27 antigen capture ELISA, where different OPV strains could be detected with sensitivities between 10^2^–10^4^ TCID_50_/mL [26, 72]. In our assay, robust detection was achieved reliably for viral loads above 1 × 10^3^ PFU/mL from cell culture supernatants for human pathogenic OPV. However, the newly described assay has the clear advantage that two mAbs are implemented as opposed to a polyclonal detection antibody. Most importantly, MPXV as the most pathogenic OPV to humankind today can be detected by both mAbs. It would be interesting to test whether mAb A3/710 could be paired with a monkeypox specific mAb described previously [70] to enable highly specific detection of MPXV with simultaneous differentiation from other OPV.

Finally, a panel of CPXV-positive samples was re-evaluated with the newly developed antigen capture ELISA. Here, samples with a high to medium viral load could be detected reliably by the antigen capture ELISA, whereas samples with Ct values above 32, indicating very low amounts of viral DNA, were not detectable. This may have been due to the presence of viral particles below the detection limit of the assay or by samples in which only viral DNA, but no virus particles, was present. However, skin lesions tested for poxviruses usually contain up to millions of virus particles. Thus, this ELISA might be superior to qPCR to identify infectious samples, whereas qPCR will be more efficient to diagnose minute amounts of virus genomes, for example when no skin lesion material is available. However, three samples that had tested highly positive by qPCR gave only very low signals at the tested 1:10 dilutions. Unfortunately, the samples could not be re-tested or tested in a plaque-forming assay due to limited amounts of sample material. Nevertheless, these results show that the ELISA could be a valuable tool for the simple detection of OPV from clinical sample material. However, further validation with a larger sample panel is needed to determine the sensitivity and specificity in a broader setting of matrices and viral loads.

## Conclusions

The newly developed mAbs and their successful implementation in an antigen capture ELISA allow for the first time the highly sensitive detection of all human pathogenic OPV tested on a particle level. These well-characterized mAbs are also promising reagents for implementation in rapid on-site detection systems like lateral flow assays [73], which could potentially fill the gap for on-site detection of VARV or MPXV in rural Africa.

## Supporting Information

S1 Fig Generation of anti A27 MAbs: successive screening for antibodies suitable for antigen capture ELISA. Scatter plots of screening and rescreening ELISA results. ELISA values for corresponding antigen pairs are plotted together. Numbers in grey boxes show percentages of clones within a specified absorption value quadrant. Green boxes mark the population of hybridoma clones that was retested in the successive ELISA (indicated by dashed grey lines). **A.** Reactivity of all hybridoma supernatants tested by screening against recombinant A27 and *E*. *coli* lysate. Almost 2/3 of all tested clones produced antibodies against A27 (OD A27 > 1.0) while only 3% reacted strongly with *E*. *coli* lysate (OD *E*. *coli* > 1.0) **B.** Cross-reactivity against His-tag (L1) and sustained antibody production as tested by reactivity against A27 during rescreening of clones with high reactivity against A27. Three main populations could be discriminated: clones that stopped production of specific antibodies (lower left box, 44.1%), clones that were cross-reactive to the His-tag (middle box and upper right box, 11.7% and 2.4%) and clones that were still highly reactive against A27 without cross-reactivity against the His-tag (green box, 29.4%). **C.** Antigen capture ELISA ability of clones reactive against A27 during rescreening. Binding of hybridoma supernatant to captured virus particles (Antigen capture VACV) and rabbit anti VACV capture antibody (Antigen capture NC) was tested. While most antibodies showed cross-reactivity against the capture antibody, eight clones (green) were reactive against captured virus particles only.
(TIF)

S2 Fig Detection of VACV-infected HEp-2 cells by anti-A27 mAbs. VACV_LE_-infected HEp-2 cells were stained with rabbit anti VACV antibodies (1:100) and FITC labelled goat anti-rabbit antibodies (1:200; green). Anti-A27 mAbs were labelled directly with DyLight647 (1:100; red). Cell nuclei were counter stained with DAPI. Scale bars = 100 μm.
(TIF)

S3 Fig Results of peptide epitope mapping of anti A27 mAbs. Shown is the mean signal intensity at 635 nm for binding of the anti A27 mAbs to spotted peptides spanning the entire A27 sequence (VACV_WR_). Recombinant A27 (BEI Resources) was included as the positive control, while an unspecific peptide (penultimate spot: GGSGGSGDYKDDDDK) was included as the negative control.
(TIF)

S4 Fig Titration series to determine the suitability of antigen capture ELISA monoclonal antibodies for OPV detection. The antibodies mentioned in the title were used for detection while antibodies mentioned in the legend were used as coating antibodies.
(TIF)

S5 Fig Results for the re-evaluation of CPXV positive clinical samples by the newly developed antigen capture ELISA. Shown are ELISA readings for 1:10 diluted sample material, tested with either the specific capture antibody A1/40 (anti Pox) or an unspecific anti-ricin capture antibody, used to account for potential unspecific binding. In seven samples, the signal intensity differed significantly between the specific and unspecific capture antibodies, whereas for three samples with Ct values above 30, no significant difference was seen. However, for three positive samples (human swab, Ct = 18.8, rat skin Ct = 14.2, human lung Ct = 10.8), ELISA signal intensities were unexpectedly low despite a high viral load indicated by qPCR. CPXV_BR_ was included as the positive control at a concentration of 10^4^ PFU/mL.
(TIF)
