# Supplementary figures and images for: Development of a Genus-Specific Antigen Capture ELISA for Orthopoxviruses – Target Selection and Optimized Screening
Source: PLoS One. 2016 Mar 1;11(3):e0150110. doi: 10.1371/journal.pone.0150110 (PMC4773239; doi:10.1371/journal.pone.0150110)

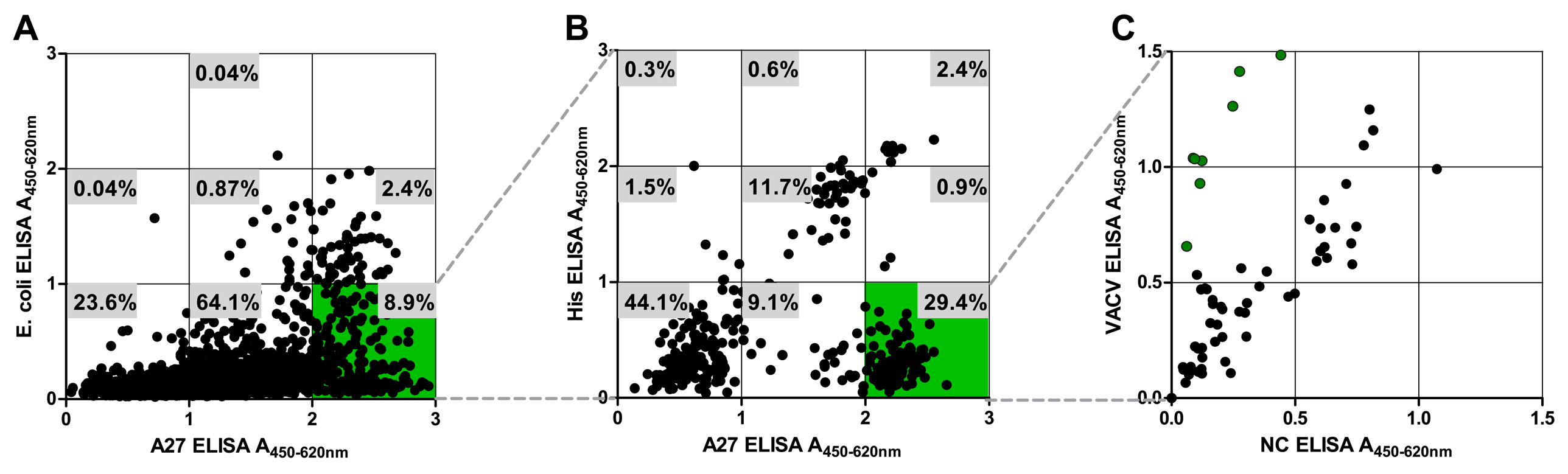

Supplement: S1 Fig — Scatter plots of screening and rescreening ELISA results. ELISA values for corresponding antigen pairs are plotted together. Numbers in grey boxes show percentages of clones within a specified absorption value quadrant. Green boxes mark the population of hybridoma clones that was retested in the successive ELISA (indicated by dashed grey lines). A. Reactivity of all hybridoma supernatants tested by screening against recombinant A27 and E. coli lysate. Almost 2/3 of all tested clones produced antibodies against A27 (OD A27 > 1.0) while only 3% reacted strongly with E. coli lysate (OD E. coli > 1.0) B. Cross-reactivity against His-tag (L1) and sustained antibody production as tested by reactivity against A27 during rescreening of clones with high reactivity against A27. Three main populations could be discriminated: clones that stopped production of specific antibodies (lower left box, 44.1%), clones that were cross-reactive to the His-tag (middle box and upper right box, 11.7% and 2.4%) and clones that were still highly reactive against A27 without cross-reactivity against the His-tag (green box, 29.4%). C. Antigen capture ELISA ability of clones reactive against A27 during rescreening. Binding of hybridoma supernatant to captured virus particles (Antigen capture VACV) and rabbit anti VACV capture antibody (Antigen capture NC) was tested. While most antibodies showed cross-reactivity against the capture antibody, eight clones (green) were reactive against captured virus particles only. (TIF) [file pone.0150110.s001.tif]

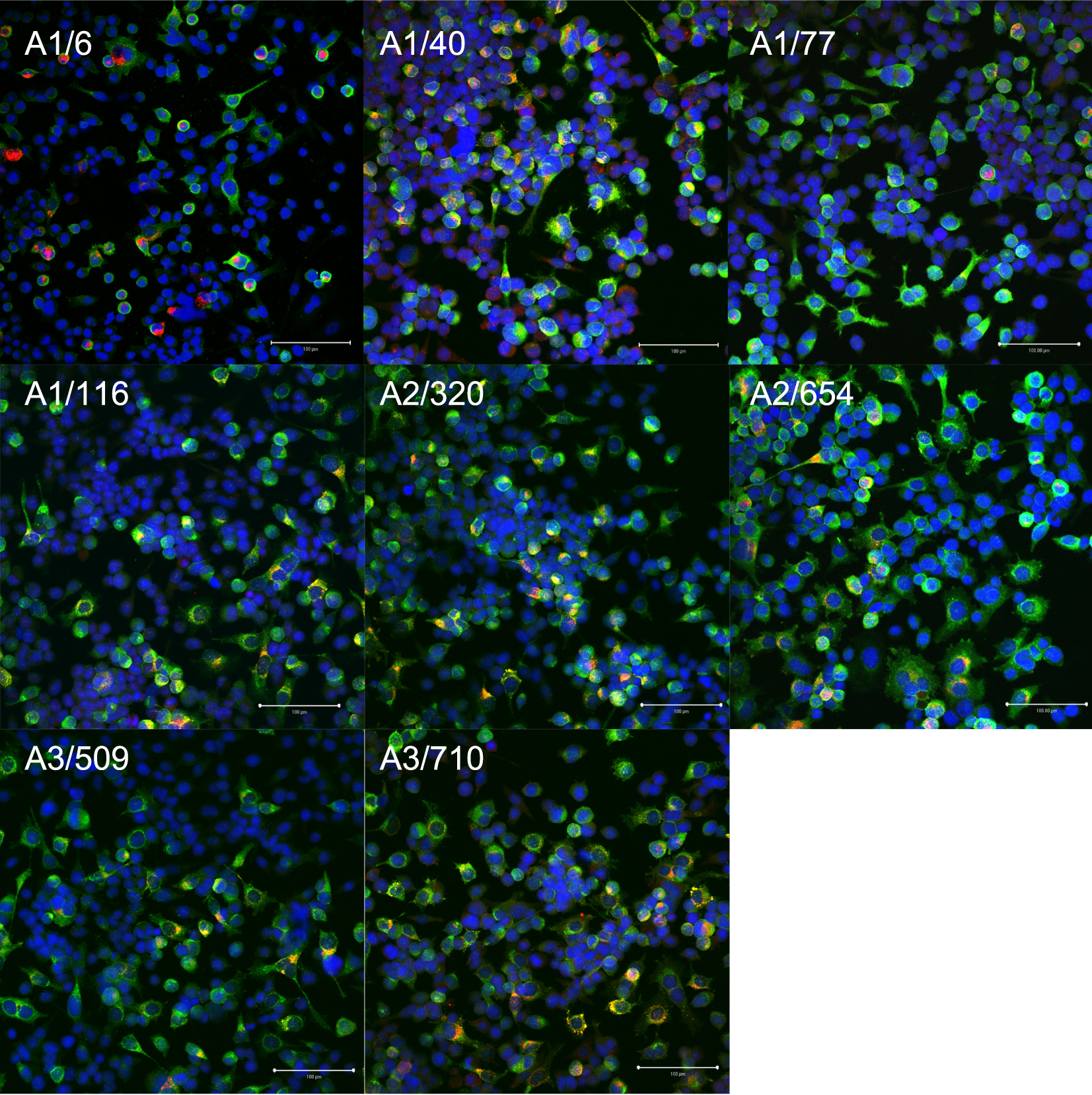

Supplement: S2 Fig — VACVLE-infected HEp-2 cells were stained with rabbit anti VACV antibodies (1:100) and FITC labelled goat anti-rabbit antibodies (1:200; green). Anti-A27 mAbs were labelled directly with DyLight647 (1:100; red). Cell nuclei were counter stained with DAPI. Scale bars = 100 μm. (TIF) [file pone.0150110.s002.tif]

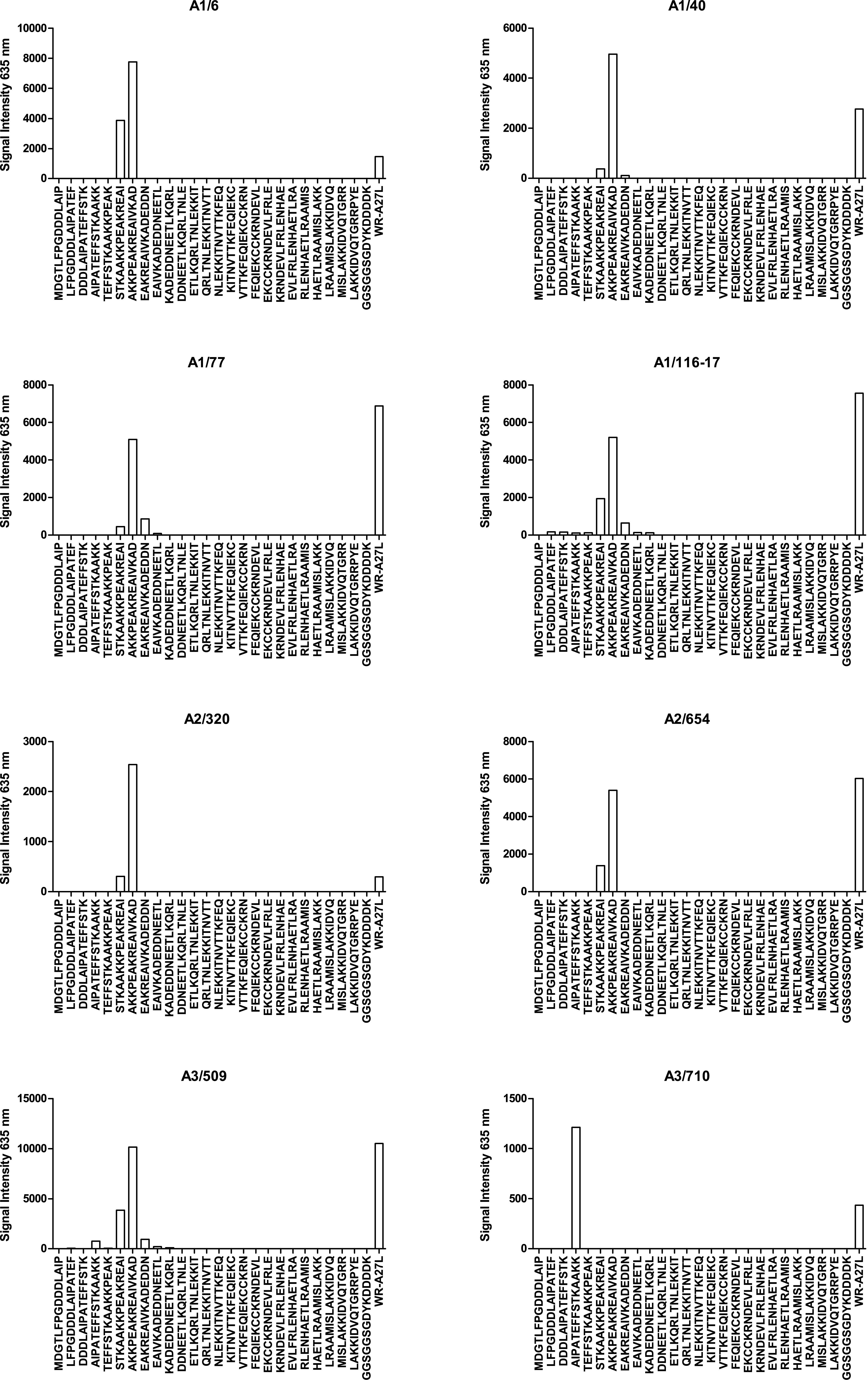

Supplement: S3 Fig — Shown is the mean signal intensity at 635 nm for binding of the anti A27 mAbs to spotted peptides spanning the entire A27 sequence (VACVWR). Recombinant A27 (BEI Resources) was included as the positive control, while an unspecific peptide (penultimate spot: GGSGGSGDYKDDDDK) was included as the negative control. (TIF) [file pone.0150110.s003.tif]

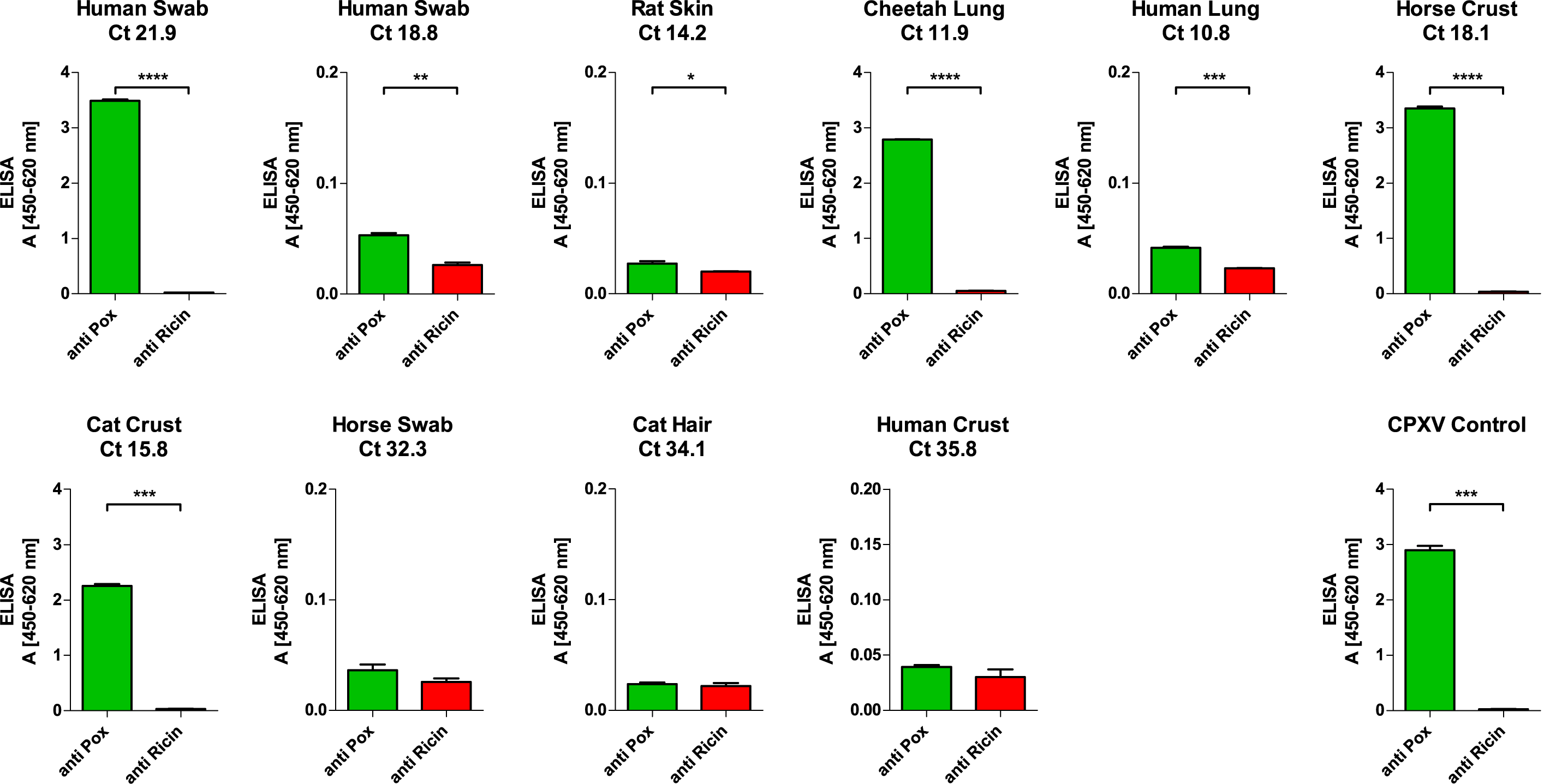

Supplement: S5 Fig — Shown are ELISA readings for 1:10 diluted sample material, tested with either the specific capture antibody A1/40 (anti Pox) or an unspecific anti-ricin capture antibody, used to account for potential unspecific binding. In seven samples, the signal intensity differed significantly between the specific and unspecific capture antibodies, whereas for three samples with Ct values above 30, no significant difference was seen. However, for three positive samples (human swab, Ct = 18.8, rat skin Ct = 14.2, human lung Ct = 10.8), ELISA signal intensities were unexpectedly low despite a high viral load indicated by qPCR. CPXVBR was included as the positive control at a concentration of 104 PFU/mL. (TIF) [file pone.0150110.s005.tif]
